# Arrival time parametric imaging of the hemodynamic balance changes between the hepatic artery and the portal vein during deep inspiration, using Sonazoid-enhanced ultrasonography: A case of Budd-Chiari syndrome

**DOI:** 10.3892/etm.2013.1105

**Published:** 2013-05-09

**Authors:** NORITAKA WAKUI, RYUJI TAKAYAMA, YASUSHI MATSUKIYO, NAOHISA KAMIYAMA, KOJIRO KOBAYASHI, TAKANORI MUKOZU, SHIGERU NAKANO, TAKASHI IKEHARA, HIDENARI NAGAI, YOSHINORI IGARASHI, YASUKIYO SUMINO

**Affiliations:** 1 Division of Gastroenterology and Hepatology, Toho University Omori Medical Center, Tokyo 143-8541;; 2 Ultrasound General Imaging, GE Healthcare, Tokyo 191-8503, Japan

**Keywords:** arrival time parametric imaging, Sonazoid-enhanced ultrasonography, peribiliary capillary plexus, Budd-Chiari syndrome, arterialization, precapillary sphincter, inspiration

## Abstract

This case report concerns a 40-year-old male who had previously been treated for an esophageal varix rupture, at the age of 30 years. The medical examination at that time revealed occlusion of the inferior vena cava in the proximity of the liver, leading to the diagnosis of the patient with Budd-Chiari syndrome. The progress of the patient was therefore monitored in an outpatient clinic. The patient had no history of drinking or smoking, but had suffered an epileptic seizure in 2004. The patient's family history revealed nothing of note. In February 2012, color Doppler ultrasonography (US) revealed a change in the blood flow in the right portal vein branch, from hepatopetal to hepatofugal, during deep inspiration. Arrival time parametric imaging (At-PI), using Sonazoid-enhanced US, was subsequently performed to examine the deep respiration-induced changes observed in the hepatic parenchymal perfusion. US images captured during deep inspiration demonstrated hepatic parenchymal perfusion predominantly in red, indicating that the major blood supply was the hepatic artery. During deep expiration, the portal venous blood flow remained hepatopetal, and hepatic parenchymal perfusion was displayed predominantly in yellow, indicating that the portal vein was the major source of the blood flow. The original diagnostic imaging results were reproduced one month subsequently by an identical procedure. At-PI enabled an investigation into the changes that were induced in the hepatic parenchymal perfusion by a compensatory mechanism involving the hepatic artery. These changes occurred in response to a reduction in the portal venous blood flow, as is observed in the arterialization of hepatic blood flow that is correlated with the progression of chronic hepatitis C. It has been established that the peribiliary capillary plexus is important in the regulation of hepatic arterial blood flow. However, this case demonstrated that the peribiliary capillary plexus also regulates acute changes in portal venous blood flow, in addition to the chronic reduction in blood flow that is observed in patients with chronic hepatitis C.

## Introduction

The liver receives a dual blood supply from the hepatic portal vein and the hepatic artery. Whilst 70–80% of the supply is provided by the portal vein, which transports nutrients and various other substances to the liver, the remaining 20–30% of the blood supply arrives via the hepatic artery, which mainly nourishes the biliary system (1). The blood pressure of the arterial system is >100 mmHg, whereas the pressure of the portal vein is considerably lower at 6–8 mmHg. As a result of this pressure difference, when problems arise in the liver or in other tissues supplied by these vessels, the portal vein is usually the primary vessel affected, which leads to a reduction in blood flow. A reduction in the total hepatic blood supply of this nature appears to activate a compensatory mechanism that increases the arterial blood flow (1–4).

Budd-Chiari syndrome occurs as a result of the congestion of the liver, due to the chronic obstruction of hepatic blood flow draining into the venous system. Long-lasting hepatic congestion has been demonstrated to lead to necrosis of the hepatocytes in the vicinity of the hepatic venules (central vein) and sinusoidal enlargement, whilst congestive cirrhosis has been indicated to develop as a result of the progressive fibrosis in the proximity of the hepatic venules (5). This leads to a further reduction in the speed and the volume of portal venous blood flow (6), eventually resulting in a cessation of the blood flow. The reduction in hepatic blood flow, due to a blockage in the portal vein, is compensated for by the hepatic arterial blood flow and the portal vein subsequently functions as a drainage vessel for the arterial blood. As a consequence, the hepatic blood flow becomes hepatofugal, and this is the mechanism for disease progression.

With the aim of gaining a better understanding the pathology of liver disease, the focus of our group is the study of hepatic hemodynamic changes. The present study concerns a case of Budd-Chiari syndrome that may reveal, in part, the mechanism that regulates the blood flow balance between the hepatic artery and the hepatic portal vein.

## Case report

### Patient history and presentation

**Figure 1.**
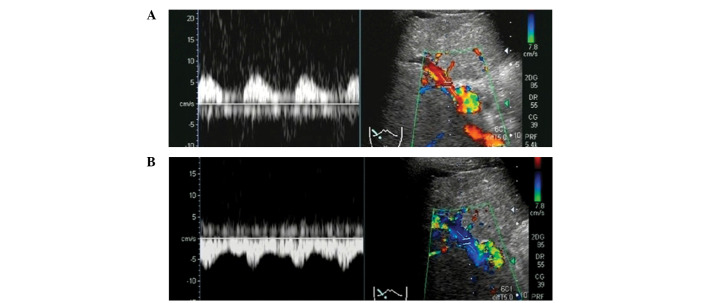
Abdominal ultrasound images. (A) Fast Fourier Transform (left) and color Doppler ultrasongraphy (US) (right) images during deep expiration reveal hepatopetal blood flow (red) in the right portal vein branch. (B) Fast Fourier Transform (left) and color Doppler US (right) images during deep inspiration demonstrate hepatofugal blood flow (blue). Images were acquired in February 2012.

**Figure 2.**
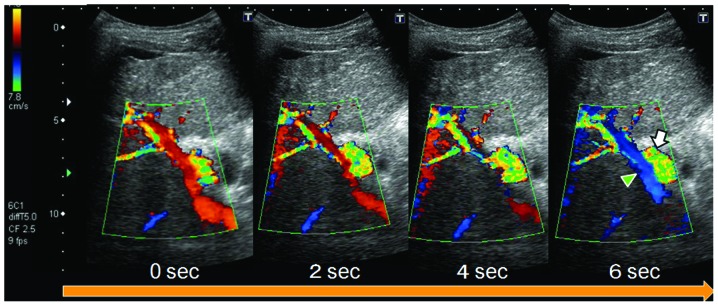
Changes in portal venous blood flow (arrow head) during deep inspiration. Color Doppler ultrasonography (US) images demonstrate the change in the blood flow from hepatopetal (red) to hepatofugal (blue) during deep inspiration breath-holding for 6 sec. The arrow indicates arterial blood flow. Images were acquired in February 2012.

A 40-year-old male had been previously admitted to The Toho University Omori Medical Center (Tokyo, Japan) for the treatment of an esophageal varix rupture, at the age of 30 years. At that time, as the physical examination indicated occlusion of the inferior vena cava in the proximity of the liver, a liver biopsy was performed. The biopsy revealed fibrosis in the vicinity of the hepatic venules (zone III), and an enlargement of the sinusoids and portal vein branches, which led to a diagnosis of Budd-Chiari syndrome. The patient was subsequently monitored in the hospital's outpatient clinic. The patient had no history of alcohol consumption or tobacco use, but had suffered a single epileptic seizure in 2004. The patient's family history revealed nothing of note. In February, 2012, the patient underwent a routine abdominal ultrasound, and the color Doppler ultrasonography (US) images revealed a change in the blood flow from hepatopetal (in red) to hepatofugal (in blue) in the right portal vein branch during deep inspiration (Figs. 1 and 2). We subsequently performed arrival time parametric imaging (At-PI), using Sonazoid-enhanced US, to investigate the balance between the blood supply from the hepatic artery and the portal vein in hepatic parenchymal perfusion during deep expiration. The patient was alert during the At-PI, and a blood pressure of 124/80 mmHg, a heart rate of 70 bpm and a body temperature of 36.0°C were recorded. The palpebral conjunctiva exhibited no signs of anemia; however, the bulbar conjunctiva demonstrated a mild yellowish discoloration. Further investigations revealed pure heart and clear breath sounds; a flat and soft abdomen with no tenderness; a palpable spleen and the absence of edema in the lower extremities. Hematological tests revealed pancytopenia and mild jaundice (Table I).

### Contrast-enhanced US and At-PI

US imaging was performed using a Toshiba Aplio XG diagnostic ultrasound system (model SSA-790A; Toshiba Medical Systems Corporation, Tochigi, Japan) with a 3.75 MHz convex array probe (model PVT-375BT, Toshiba Medical Systems Corporation) at a mechanical index of 0.21. The right main branch of the portal vein was visualized from the right intercostal space, and images displaying the liver parenchyma of the right hepatic lobe (segments 5–8) were selected for analysis. The focal depth was set at 3–10 cm using the dual-focus mode. Subsequent to the selection of the imaging parameters, the recommended dose of Sonazoid (perfluorobutane; 0.015 ml/kg), obtained from GE Healthcare (Oslo, Norway), was injected via the cubital vein. Following the Sonaziod infusion, imaging was performed for ∼40 sec to visualize the patient during a period of deep inspiration, and the images were then stored as raw data in the system hardware. The images corresponding with deep expiration were acquired in the same manner. US was performed by the same operator each time to maintain imaging consistency.

**Figure 3.**
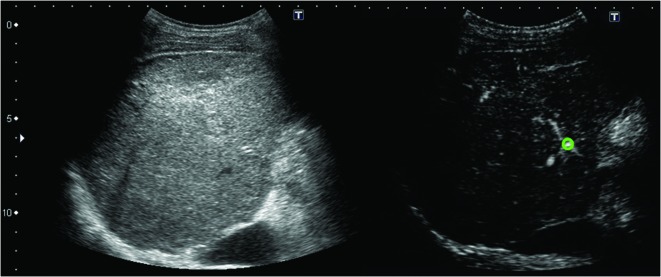
Arrival time parametric imaging (At-PI): Left, B-mode ultrasonography (US) image; right, a Sonazoid-enhanced US image. The region of interest (green circle) was set on the large hepatic artery branch in the right hepatic hilum.

**Figure 4.**
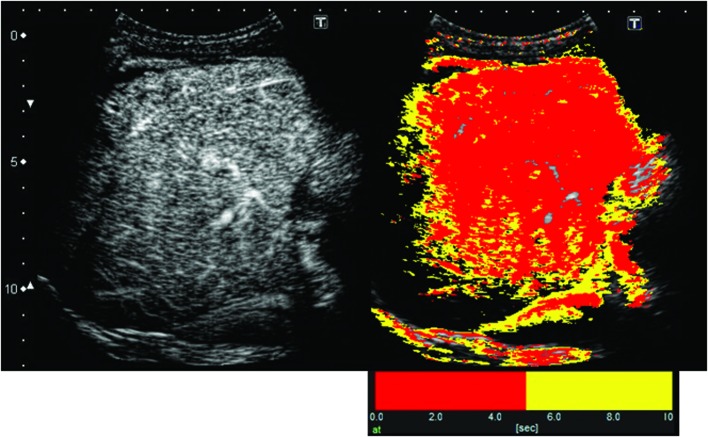
Contrast enhancement pattern of the liver parenchyma 10 sec subsequent to the arrival of the contrast medium, following a bolus infusion of Sonazoid via the median cubital vein: Sonazoid-enhanced images of the liver parenchyma with (right) and without (left) arrival time parametric imaging (At-PI).

**Figure 5.**
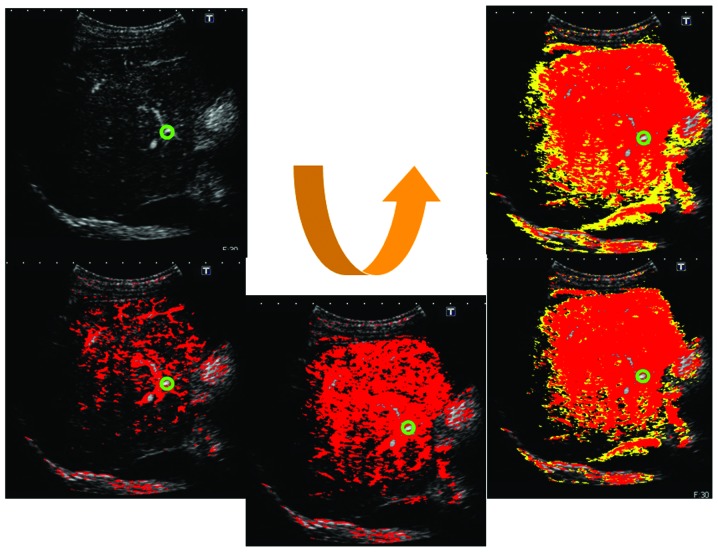
Creating arrival time parametric imaging (At-PI) images. With the large hepatic artery branch in the right hepatic hilum as the region of interest (ROI; the green circle), the ultrasonography system set the point at which the ROI was contrasted as time 0, sequentially calculated the arrival time of individual pixels in the hepatic parenchyma and automatically color coded B mode images. Red pixels indicate an arrival time of 0–5 sec, and yellow pixels indicate an arrival time of 6–10 sec.

At-PI images were generated from the stored data using the software interfaced with the ultrasound system. The right branch of the hepatic artery was selected as the region of interest (ROI); thus, the system set the moment at which the ROI was contrasted as time 0, and sequentially calculated the arrival time of individual pixels in the hepatic parenchyma. The system subsequently automatically created and superimposed a color map onto a B-mode image. In the present study, it took ∼10 sec for the contrast agent to completely visualize the liver parenchyma, following its arrival at the hepatic artery. We therefore displayed pixels arriving between 0–5 sec in red, and those arriving between 6–10 sec in yellow (Figs. 3–5).

### Calculation of the ratio of the area of red pixels to the entire contrast-enhanced area

**Figure 6.**
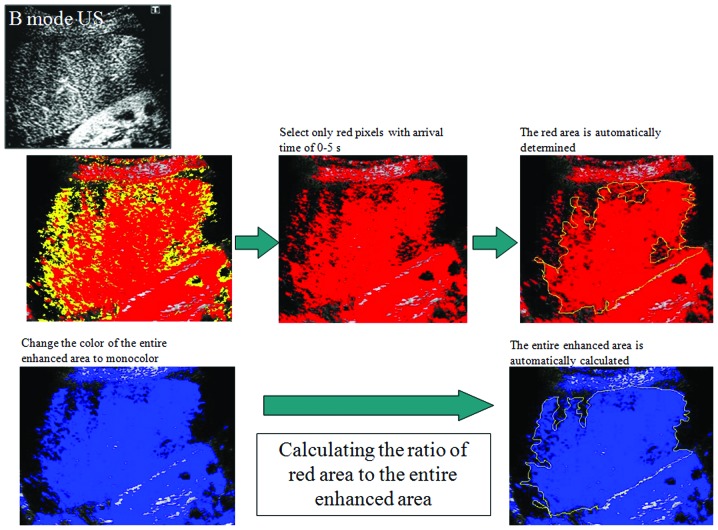
Procedure for calculating the ratio of the area of red pixels to the entire contrast-enhanced area of the liver parenchyma. From the obtained arrival time parametric imaging (At-PI) images, the red area of the liver parenchyma and the entire contrast-enhanced area were calculated using ImageJ software (version 1.42; Rasband WS, US National Institutes of Health), in order to determine the ratio of red (ROR).

For the quantitative evaluation of the At-PI data obtained, the ratio of the area of red pixels, i.e. the pixels with shorter arrival times, to the entire contrast-enhanced area was calculated as the ‘ratio of red’ (ROR), using the image analysis software ImageJ (version 1.42; Rasband WS, US National Institutes of Health, Bethseda, MD, USA; Fig. 6).

### Imaging and calculation results

**Figure 7.**
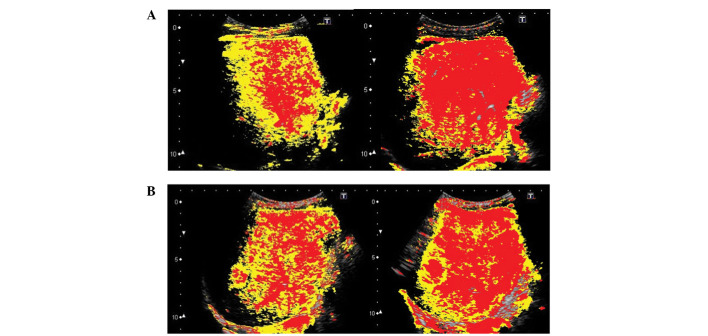
Arrival time parametric imaging (At-PI) during deep inspiration (right) and deep expiration (left). (A) At-PI images demonstrating an ROR for deep inspiration of 77.1% and for deep expiration of 44.5%. Images were acquired in February 2012. (B) At-PI images demonstrating an ROR for deep inspiration of 76.9% and for deep expiration of 56.3%. Images were acquired in March 2012.

In the first US imaging, conducted in February 2012, the ROR for deep inspiration was 77.1% and that of deep expiration was 44.5%. In the second imaging procedure, conducted in March 2012, the ROR for deep inspiration was 76.9% and that of deep expiration was 56.3%. These results demonstrated that the ROR was higher for deep inspiration than expiration (Fig. 7).

## Discussion

At-PI, a US image analysis tool that was introduced into the Toshiba Aplio XG diagnostic ultrasound system (Toshiba Medical Systems Corporation) in October 2010, traces and color codes temporal changes in contrast-enhanced US images. We have previously used At-PI to investigate the influence of the hepatic portal vein and the hepatic artery on hepatic parenchymal enhancement, and have reported its use in the clinical assessment of type C chronic liver disease (7) and alcohol-induced liver disease (8). We have also utilized At-PI to investigate the effects of portal vein thrombosis on hepatic parenchymal enhancement (9), demonstrating its efficacy in the differential diagnosis of benign recurrent intrahepatic and familial intrahepatic cholestasis (10).

In the present study, At-PI technology was used to reveal changes in hepatic parenchymal enhancement in a patient whose blood flow balance between the hepatic artery and portal vein was affected by deep breathing. The arrival time of Sonazoid in the liver was the primary element that was considered in the study of this blood flow balance. The contrast agent, injected via the cubital vein, first arrived at the liver via the hepatic artery, having passed through the heart. It was also carried through the venous system to arrive at the liver via the portal vein, once the blood had first nourished the stomach, intestines and spleen. The contrast agent has been demonstrated to arrive at the liver via these two routes ∼5 sec apart, with the hepatic arterial blood flow arriving more rapidly (11). In At-PI, blood flow arriving 0–5 sec following the contrast agent injection is displayed in red, and is from the hepatic artery, whereas blood flow arriving 6–10 sec subsequent to the injection is displayed in yellow, and is from the portal vein. The system therefore enables an objective study of the blood flow balance between the hepatic artery and the portal vein.

Budd-Chiari syndrome occurs as a result of the congestion of the liver, due to the chronic obstruction of hepatic blood flow draining into the venous system. Long-lasting hepatic congestion has been demonstrated to lead to necrosis of the hepatocytes in the vicinity of the hepatic venules (central vein), and sinusoidal enlargement, whilst congestive cirrhosis has been indicated to develop as a result of the progressive fibrosis in the proximity of the hepatic venules (5). This leads to a further reduction in the speed and the volume of portal venous blood flow (6), eventually resulting in a cessation of the blood flow. The reduction in hepatic blood flow, due to a blockage in the portal vein, is compensated for by the hepatic arterial blood flow, and the portal vein subsequently functions as a drainage vessel for the arterial blood. As a consequence, the hepatic blood flow becomes hepatofugal, and this is the mechanism for disease progression. Several studies have investigated the effect of respiration on hepatic blood flow, and have demonstrated that portal venous blood flow is reduced by inspiration and enhanced by expiration (12–15), due to an increase in the intra-abdominal pressure. This occurs since inspiration compresses the blood vessels in the liver, and increases the resistance to portal venous blood flow (12). In the present study, the change in portal venous blood flow occurred as a result of deep inspiration, due to a slight increase in the intra-abdominal pressure, which indicated that at this time the portal venous blood flow of the patient was changing from hepatopetal to hepatofugal.

As observed in the present case, when the portal venous blood flow is reduced, the hepatic vascular system has a mechanism to compensate for the loss in hepatic parenchymal perfusion (1–4). However, prior to this case, it had not been determined whether the hepatic artery is able to fully respond to acute changes in portal venous blood flow. Therefore, this was investigated in the present study, using At-PI. In deep inspiration, when the portal venous blood flow was hepatofugal, the hepatic parenchymal perfusion was displayed by early-arriving red pixels, indicating that hepatic arterial blood flow was the major source of blood flow. However, during deep expiration, when the portal venous blood flow was hepatopetal, there was a reduction in the proportion of red pixels in the hepatic parenchymal perfusion, indicating an increase in portal venous blood flow. These results appeared to indicate that the hepatic artery successfully compensated for the acute reduction in the portal venous blood flow.

Blood flow in the liver, particularly intrahepatic micro-circulation, is important in the study of changes in hepatic parenchymal perfusion. Sonazoid, injected via the cubital vein, arrives at the liver via arterial blood flow. In healthy individuals, the hepatic artery is the blood vessel that feeds the biliary system. Arterial blood flow nourishes large and small bile ducts, while traveling through the liver toward the periphery. The peribiliary capillary plexus is formed around the bile ducts, and a number of the capillaries merge into the terminal portal venules and sinusoids. However, certain branches of the hepatic artery merge directly into the sinusoids, without passing through the peribiliary capillary plexus, or nourish the portal vein by extending into the wall (16–18).

In healthy individuals, the ratio of blood inflow via the hepatic portal vein and the hepatic artery is 7–8:2–3, and therefore the portal vein is the predominant blood supply to the liver (1). This is considered to be due to the fact that blood flowing through the portal vein contains nutrients from the stomach and the intestine, which results in the portal vein acting as a nutrient vessel for the liver. A previous study, which investigated the pressure difference between the two vascular systems by micropuncturing the hepatic microvasculature under a biomicroscope, determined that blood pressure at the distal end branches of the hepatic artery was 6–8-fold greater than that at the portal vein branches (300–400 vs. 50 mm H_2_O, respectively) (19). The ability of the portal vein to carry large volumes of blood into the sinusoids at this low pressure is, in part, due to the precapillary sphincter. It has been demonstrated that the distal end branches of the hepatic artery and the peribiliary capillary plexus around the bile ducts exist in the form of capillaries, and that each capillary is encircled by a precapillary sphincter. This sphincter adjusts the blood flow from the arterioles into the capillary (20), thus controlling the volume of blood entering the capillary from the high-pressure arterial system. The pumping of the blood into the sinusoids by the low-pressure portal vein system is therefore facilitated by the precapillary sphincter.

The investigations in the present study demonstrated that, during deep inspiration, when portal venous blood flow was obstructed by an increase in the intra-abdominal pressure, the reduction in hepatic parenchymal perfusion was immediately counterbalanced by the hepatic artery. As a result of this compensatory mechanism, the portal vein appeared to serve as a drainage vessel for the hepatic artery, and changed the blood flow to hepatofugal. The reduction in the portal venous blood flow may have generated signals to stimulate the precapillary sphincter, which then regulated hepatic parenchymal perfusion by activating an alternative mechanism that enhanced hepatic arterial blood flow into the sinusoids. During expiration, when intra-abdominal pressure was reduced, blood flow through the low-pressure portal vein into the liver parenchyma was facilitated. Consequently, the precapillary sphincter regulated the hepatic arterial blood flow to reestablish the dominance of the portal vein in the hepatic hemodynamics. In the present study, it was possible to establish how hepatic parenchymal perfusion was affected by acute changes in the portal venous blood flow by examining a patient with Budd-Chiari syndrome, who exhibited an inspiration-induced change in the portal venous blood flow, from hepatopetal to hepatofugal. At-PI was utilized as a simple and effective imaging tool for the visualization of these changes in hepatic hemodynamics.

Due to the lack of valves, blood flow in the hepatic portal vein is easily converted to hepatofugal blood flow, as the pressure gradient of hepatic parenchymal perfusion is reversed by a change in the hemodynamics. Comprehensive studies of hepatofugal blood flow in the portal venous system, particularly in the main portal vein, have demonstrated that the reversal of portal venous blood flow is rare when the portosystemic shunt is naturally occurring. It is possible that this is due to the fact that hepatic blood flow is maintained by a mechanism that prevents portal venous pressure from decreasing below sinusoidal pressure (21–29). In the case of hepatofugal portal venous blood flow, the mechanism that maintains hepatopetal blood flow may be impaired due to obstructed hepatic outflow, a characteristic of Budd-Chiari syndrome. This may be in addition to the increased sinusoidal pressure that occurs as a result of a hepatocellular carcinoma-induced hepatic arterial-portal venous shunt (23,27). However, a study by Nishida *et al* indicated that the conversion of hepatic blood flow from hepatofugal to hepatopetal occurred following transcatheter arterial embolization therapy (TAE) for hepatocellular carcinoma (30). This may have been a result of the closure of the hepatic arterial-portal venous shunt due to the TAE, leading to a reduction in the arterial blood flow and the reestablishment of hepatopetal blood flow in the portal vein.

To the best of our knowledge, the present study is the first in which respiration-induced reversible changes in hepatic blood flow, from hepatopetal to hepatofugal, have been investigated with the use of the At-PI technology. The results have provided an insight into the effect of respiration on the hemodynamic changes between the hepatic artery and portal vein.

We have previously used At-PI to investigate the hepatic arterial dominance that occurs as a result of disease progression in patients with chronic hepatitis C (7). The peribiliary capillary plexus has been demonstrated to be important in the compensatory mechanism of the hepatic artery that counteracts any reduction in portal venous blood flow. In the present study, we revealed that the peribiliary capillary plexus also regulated the acute reduction in portal venous blood flow, in addition to the chronically progressive reduction that is apparent in patients with hepatitis C.

## Figures and Tables

**Table I.** Test results for a patient with Budd-Chiari syndrome.

Type of test	Result
Biochemical	
CRP (mg/dl)	1.1
Na (mEq/l)	139.0
K (mEq/l)	3.3
Cl (mEq/l)	103.0
TP (g/dl)	7.9
Alb (g/dl)	3.6
T-Bil (mg/dl)	2.8
D-Bil (mg/dl)	1.7
AST (IU/l)	28
ALT (IU/l)	14
LDH (IU/l)	189
ALP (IU/l)	574
γ-GTP (IU/l)	166
BUN (mg/dl)	7.00
Cr (mg/dl)	0.46
BS (mg/dl)	122.00
PT (%)	56
PT-INR	1.5
NH_3_ (*μ*g/dl)	76
Hematogical	
WBC (per *μ*l)	1.9×10^3^
RBC (per *μ*l)	4.66×10^6^
Hgb (mg/dl)	9.5
Hct (%)	30.7
PLT (per *μ*l)	6.5×10^4^
Serological	
HCV-Ab (+/-)	(-)
HBs-Ag (+/-)	(-)
HBs-Ab (+/-)	(-)
ANA (+/-)	(-)
AMA (+/-)	(-)
P-ANCA (+/-)	(-)
IgG (mg/dl)	981
IgA (mg/dl)	263
IgM (mg/dl)	97

CRP, C-reactive protein; Na, sodium; K, potassium; Cl, chloride; TP, total protein; Alb, albumin; T-Bil, total bilirubin, D-Bil, direct bilirubin; AST, aspartate aminotransferase; ALT, alanine aminotransferase; LDH, lactate dehydrogenase; ALP, alkaline phosphatase; GTP, glutamyl transpeptidase; BUN, blood urea nitrogen; Cr, creatinine; BS, blood sugar; PT, prothrombin time; PT-INR, prothrombin time-international normalized ratio; NH_3_, ammonia; WBC, white blood cell; RBC, red blood cell; Hgb, hemoglobin; Hct, hematocrit; PLT, platelets; HCV-Ab, hepatitis C antibody; HBs-Ag, hepatitis B surface antigen; HBs-Ab, hepatitis B surface antibody; ANA, anti-nuclear antibody; AMA, antimitochondrial antibody; P-ANCA, peripheral antineutrophil cytoplasmic antibody; IgG, immunoglobulin G; IgA, immunoglobulin A; IgM, immunoglobulin M.

## Abbreviations:

At-PI: arrival time parametric imaging;
US: ultrasonography;
ROI: region of interest;
ROR: ratio of red

## References

[1] Kleber G, Steudel N, Behrmann C (1999). Hepatic arterial flow volume and reserve in patients with cirrhosis: use of intra-arterial Doppler and adenosine infusion. Gastroenterology.

[2] Rocheleau B, Ethier C, Houle R, Huet PM, Bilodeau M (1999). Hepatic artery buffer response following left portal vein ligation: its role in liver tissue homeostasis. Am J Physiol.

[3] Leen E, Goldberg JA, Anderson JR (1993). Hepatic perfusion changes in patients with liver metastases: comparison with those patients with cirrhosis. Gut.

[4] Lautt WW (1985). Mechanism and role of intrinsic regulation of hepatic arterial blood flow: hepatic arteial buffer response. Am J Physiol.

[5] Menon KV, Shah V, Kamath PS (2004). The Budd-Chiari syndrome. N Engl J Med.

[6] Iwao T, Toyonaga A, Oho K (1997). Value of Doppler ultrasound parameters of portal vein and hepatic artery in the diagnosis of cirrhosis and portal hypertension. Am J Gastroenterol.

[7] Wakui N, Takayama R, Kanekawa T (2012). Usefulness of arrival time parametric imaging in evaluating the degree of liver disease progression in chronic hepatitis C infection. J Ultrasound Med.

[8] Wakui N, Takayama R, Mimura T (2011). Drinking status of heavy drinkers detected by arrival time parametric imaging using Sonazoid-enhanced ultrasonography: study of two cases. Case Rep Gastroenterol.

[9] Wakui N, Takayama R, Matsukiyo Y (2013). Visualization of segmental arterialization with arrival time parametric imaging using Sonazoid-enhanced ultrasonography in portal vein thrombosis: A case report. Exp Ther Med.

[10] Wakui N, Fujita M, Oba N (2013). Endoscopic nasobiliary drainage improves jaundice attack symptoms in benign recurrent intrahepatic cholestasis: A case report. Exp Ther Med.

[11] Shunichi S, Hiroko I, Fuminori M, Waki H (2009). Definition of contrast enhancement phases of the liver using a perfluoro-based microbubble agent, perflubutane microbubbles. Ultrasound Med Biol.

[12] Smith HJ, Grøttum P, Simonsen S (1985). Ultrasonic assessment of abdominal venous return. I Effect of cardiac action and respiration on mean velocity pattern, cross-sectional area and flow in the inferior vena cava and portal vein. Acta Radiol Diagn (Stockh).

[13] Moreno AH, Burchell AR, Van der Woude R, Burke JH (1967). Respiratory regulation of splanchnic and systemic venous return. Am J Physiol.

[14] Rabinovici N, Navot N (1980). The relationship between respiration, pressure and flow distribution in the vena cava and portal and hepatic veins. Surg Gynecol Obstet.

[15] Sugano S, Yamamoto K, Sasao K, Watanabe M (1999). Portal venous blood flow while breath-holding after inspiration or expiration and during normal respiration in controls and cirrhotics. J Gastroenterol.

[16] Rappaport AM, Black RG, Lucas CC, Ridout JH, Best CH (1966). Normal and pathologic microcirculation of the living mammalian liver. Rev Int Hepatol.

[17] Rappaport AM (1980). Hepatic blood flow: morphologic aspects and physiologic regulation. Int Rev Physiol.

[18] Ekataksin W, Kaneda K (1999). Liver microvascular architecture: an insight into the pathophysiology of portal hypertension. Semin Liver Dis.

[19] Nakata K, Leong GF, Brauer RW (1960). Direct measurement of blood pressures in minute vessels of the liver. Am J Physiol.

[20] Rhodin JA (1967). The ultrastructure of mammalian arterioles and precapillary sphincters. J Ultrastruct Res.

[21] Tochio H, Kudo M, Nishiuma S, Okabe Y (2001). Intrahepatic spontaneous retrograde portal flow in patients with cirrhosis of the liver: reversal by food intake. AJR Am J Roentgenol.

[22] Kessler RE, Tice DA, Zimmon DS (1969). Retrograde flow of portal vein blood in patients with cirrhosis. Radiology.

[23] Okuda K, Moriyama M, Yasumoto M, Jinnouchi S, Shimokawa Y (1973). Roentgenologic demonstration of spontaneous reversal of portal blood flow in cirrhosis and primary carcinoma of the liver. Am J Roentgenol Radium Ther Nucl Med.

[24] Foster DN, Herlinger H, Miloszewski KJ, Losowsky MS (1978). Hepatofugal portal blood flow in hepatic cirrhosis. Ann Surg.

[25] Takayasu K, Takashi M, Musha H (1982). Spontaneous reversal of portal blood flow demonstrated by percutaneous transhepatic catheterization: report of two cases. Gastroenterology.

[26] Sarfeh IJ, Rypins EB, Conroy RM, Mason GR (1983). Portacaval H-graft: relationships of shunt diameter, portal flow patterns and encephalopathy. Ann Surg.

[27] Tanabe T, Tobe K, Koide N (1985). Blood flow dynamics of the portal venous system in liver diseases studied by the ultrasonic pulsed doppler method. Kanzo.

[28] Ohnishi K, Saito M, Sato S (1985). Direction of splenic venous flow assessed by pulsed Doppler flowmetry in patients with a large splenorenal shunt. Relation to spontaneous hepatic encephalopathy. Gastroenterology.

[29] Kawasaki T, Moriyasu F, Nishida O (1987). Analysis of hepatofugal flow in portal venous system using ultrasonic Doppler duplex system. Nihon Shokakibyo Gakkai Zasshi.

[30] Nishida O, Nishikawa K, Seko S (1997). Two cases of reverse portal blood flow: clinical course and changes in hemodynamics. Journal of Medical Ultrasonics.

